# Development of a Flow Injection System for Differential Pulse Amperometry and Its Application for Diazepam Determination

**DOI:** 10.1155/2018/6121489

**Published:** 2018-03-19

**Authors:** Vesna Antunović, Slavna Tešanović, Danica Perušković, Nikola Stevanović, Rada Baošić, Snežana Mandić, Aleksandar Lolić

**Affiliations:** ^1^Faculty of Medicine, University of Banja Luka, 78000 Banja Luka, Bosnia and Herzegovina; ^2^Faculty of Chemistry, University of Belgrade, 11000 Belgrade, Serbia

## Abstract

This work presents the development of a flow injection system for differential pulse amperometry (DPA) for diazepam determination in the presence of oxygen. The thin flow cell consisted of the bare glassy carbon electrode, reference silver/silver chloride, and stainless steel as the auxiliary electrode. Electrochemical reduction of diazepam (DZP) was characterised by cyclic voltammetry. Azomethine reduction peak was used for DZP quantification. The detector response was linear in the range 20–250 *µ*mol/dm^3^ of diazepam, with a calculated detection limit of 3.83 *µ*g/cm^3^. Intraday and interday precision were 1.53 and 10.8%, respectively. The method was applied on three beverage samples, energetic drink, and two different beer samples, and obtained recoveries were from 93.65 up to 104.96%. The throughoutput of the method was up to 90 analyses per hour.

## 1. Introduction

Amperometric detection can operate in three different modes: constant potential, pulse mode, and differential pulse (or multi pulse mode). Detection at constant potential is usually applied with flow injection analysis systems because simple and cheap potentiostats can be used. When an analyte passes through the detector, it generates a current proportional to the analyte concentration [1].

Differential pulse amperometry (DPA) is a technique described and first applied by Marcenac and Gonon in 1985 [2]. In DPA, a clean potential is applied for electrode cleaning without current sampling. Then two potential pulses are applied after the cleaning step, and the current at the end of each pulse is recorded as the function of time. During the experiment, only the difference of two current samples is displayed. Obtained dependence of Δ*I* (the difference of the currents recorded at the end of each potential pulse) with time is named differential pulse amperogram [3]. The main advantage of DPA is that its signal is less affected by interferences than with the other amperometric modes.

Throughout the years, DPA has been used for determination of various substances in a flow injection (FI) system. Quadrupole-pulsed amperometric detection was applied for simultaneous determination of glucose and fructose in a FI system [4]. The first pulse was used for the oxidation of glucose and the second for both analytes. The subtraction of obtained currents was used for quantification of fructose. Three-pulse FI-DPA was used for simultaneous determination of phenolic antioxidants (butylated hydroxyanisole and butylated hydroxytoluene) at a boron-doped diamond electrode [5]. Multiple-pulse amperometric flow injection system was described for simultaneous determination of pharmaceutical active substances, paracetamol and ascorbic acid [6], caffeine, ibuprofen, and paracetamol [7], and dypirone and paracetamol in pharmaceutical formulations on a bare glassy carbon electrode (GCE) [8]. A simple, fast and low-cost multiple-pulse amperometric FI system was applied for determination of sildenafil citrate (SC) in Viagra [9]. Three sequential potential pulses were applied as a function of time. SC is detected at two different irreversible oxidation processes (at 1.6 and 1.9 V), and the third pulse (1.0 V) was used for regeneration of the surface of the boron-doped diamond electrode.

Diazepam (DZP) (7-chloro-1,3-dihydro-1-methyl-5-phenyl-2*H*-1,4-benzodiapin-2-one) belongs to the group of 1,4-benzodiazepines. It is one of the most prescribed 1,4-benzodiazepines for a number of conditions such as anxiety, insomnia, epilepsy, and muscular spasms [10]. Its low price and availability on the black market increase the risk of its abuse. Since it is highly liposolubile, it distributes rapidly inside tissues and has the high absorption rate. The absorption rate is increased when it is mixed with alcohol which enhances its sedative effect [11]. That is why diazepam is well known as “date rape” drug, and it is classified as a drug-facilitated crimes (DFC) drug [12]. A fast and simple system for diazepam determination would be of great importance for forensic science and resolving criminal activities which are related with benzodiazepine drug abuse [13].

Determination of benzodiazepines is well documented in the literature, and various spectrophotometric [14] and electrochemical detectors [15–20] were employed. Mass spectrometers were also applied to benzodiazepine determination [21]; their wide linear ranges and low detection limits are advantageous but are still expensive compared to simple flow injection systems.

Although there are many references describing determination of benzodiazepine by electrochemical detectors, there is no paper of DPA application on diazepam determination.

Electrochemical determination of diazepam is based on reduction of 4,5-azomethine group. This simple reduction yielding dihydro specie is used for quantification of diazepam [22]. Applying more negative potentials increases interferences from oxygen, hydrogen ions, and other metal ions present in the sample [23]. Due to high background currents, sensitive determination is almost impossible. Concentration of hydrogen and metal ions is a matter of optimization process and can be controlled. However, the presence of oxygen is more problematic and can be decreased either by removing with nitrogen or by applying modifications of electrode surfaces. Often purging of nitrogen or argon prior the analyses for a few minutes or even overnight is usually enough to remove dissolved oxygen from working solutions [23]. When flow injection systems are applied, another problem arises: the choice of tubing material. Usually, standard Teflon tubings should be avoided due to absorption of oxygen through the polymer. Other polymer materials are more or less chemically inert and also affect the price of the flow system or even using glass or metallic materials but they are too rigid for handling the simple flow system. Lozano-Chaves [15] applied modified carbon-paste electrodes as sensors for the determination of diazepam and its metabolites temazepam and oxazepam in biological fluids. As a modifier, they used 5% bentonite and 5% zeolite. Bentonite-modified electrodes showed better sensitivities. Diazepam was determined at pH 10 in Britton–Robinson buffer, with the concentration range of 0.025–3.0 mg/dm^3^ and the detection limit of 0.021 mg/dm^3^; all experiments were performed under nitrogen atmosphere. Multiwall carbon nanotube-ionic liquid modified paste electrode was used for diazepam determination in real samples [24]. Analytical parameters for diazepam determination were linearity in the range of 0.02–0.76 mg/dm^3^ and the detection limit of 4.1 *µ*g/dm^3^. Experiments were performed under nitrogen atmosphere. As mentioned earlier, reduction of 4,5-azomethine group is often used for quantification of diazepam. Group of authors [20] noticed that there is an oxidation peak in a reverse scan in cyclic voltammetry and they determined the oxidation potential (+1.0 V versus Ag/AgCl) for diazepam in drinks on a screen-printed electrode by adsorptive stripping voltammetry. The detector response was linear in the range of 7.1–285 mg/dm^3^ and a detection limit of 1.8 mg/dm^3^.

This work presents the development of a flow injection for diazepam determination with differential pulse amperometry and its application in spiked beverage samples without removal of dissolved oxygen.

## 2. Materials and Methods

### 2.1. Reagents and Chemicals

All reagents used were of analytical grade quality, and all solutions were prepared in degassed and filtered deionised water. Buffer solution of HCl/KCl pH 1 was prepared by mixing appropriate volumes of the diluted solution of the hydrochloric acid (0.2 mol/dm^3^, Carlo Erba, Val de Reuil, France) and potassium chloride (0.2 mol/dm^3^, Betahem, Belgrade, Serbia). Britton-Robinson buffer was prepared by mixing 0.04 mol/dm^3^ CH_3_COOH (Carlo Erba, Val de Reuil, France), 0.04 mol/dm^3^ H_3_PO_4_ (Carlo Erba, Val de Reuil, France), and 0.04 mol/dm^3^ H_3_BO_3_ (Betahem, Belgrade, Serbia), and the pH value is adjusted with 0.02 mol/dm^3^ NaOH (Betahem, Belgrade, Serbia). The stock solution of diazepam (Hemofarm, Vršac, Serbia) was prepared by dissolving the required mass in methanol to give a concentration of 10 mmol/dm^3^. Stock solution was maintained under refrigerated conditions in the absence of light, and it was prepared once a week. Working standards were prepared daily by dilution of this solution with appropriate buffers.

### 2.2. Apparatus

Cyclic voltammetry was used for electrochemical study of diazepam. For the cyclic voltammetry, a CHI 800C potentiostat was used. A three-electrode system consisted of the glassy carbon working electrode (CH Instruments, USA, model CHI104, 3 mm in diameter), Ag/AgCl reference electrode (CH Instruments, USA; CHI111), and platinum wire as auxiliary electrode. pH measurements were performed using WTW720 pH-meter equipped with SenTix 81 pH electrode. Prior the cyclic voltammetry experiments, all solutions were purged with oxygen free nitrogen for 15 minutes.

Cleaning of glassy carbon electrodes was performed mechanically on a polishing pad using aluminium paste of different grain sizes (1, 0.3, and 0.05 *µ*m, Buehler, USA). It was washed with distilled water between each paste, and after the finest paste, it was washed with methanol, distilled water, and then air-dried. This process was repeated each day before the start of the recording and in the case if detector response was not reproductive.

### 2.3. Flow Injection Analysis System

Peristaltic pump model Mini S 840 (Ismatec, Switzerland) was used. Two-position injection valve model 5020 (Rheodyne, USA) equipped with a sample loop was used. Amperometric flow cell (thin layer, BASi, USA) consists of working, reference, and auxiliary electrodes [25]. The working electrode was glassy carbon electrode (BAS Instruments, model MF-1008, USA) consisting of two circular, dual series electrodes which are embedded in a polymer based on fluorocarbons. The reference Ag/AgCl (BAS Instruments, model MF-2021, USA) electrode is filled with 3 mol/dm^3^ NaCl solution, and the auxiliary electrode is made of stainless steel. Teflon gasket was placed between the auxiliary and working electrodes; its thickness regulates the volume of the working solution. Electrochemical measurements were performed on model CHI760b. The CHI software was used for data acquisition.

### 2.4. Electrochemical Procedure

Each DPA cycle applied to the working electrode consisted of three steps: first step, cleaning potential of +0.7 V for 50 ms, second −0.75 V for 50 ms (first potential pulse), and then −0.95 V for 50 ms (second potential pulse). The analytical signal was calculated as the difference between the average signal at last 25 ms of the second and the first potential pulse. DPA measurements were performed on CHI760b potentiostat. For this set of experiments, there was no need to expel the oxygen from solutions.

## 3. Results and Discussion

### 3.1. Cyclic Voltammetry of Diazepam and pH Effect

**Figure 1:**
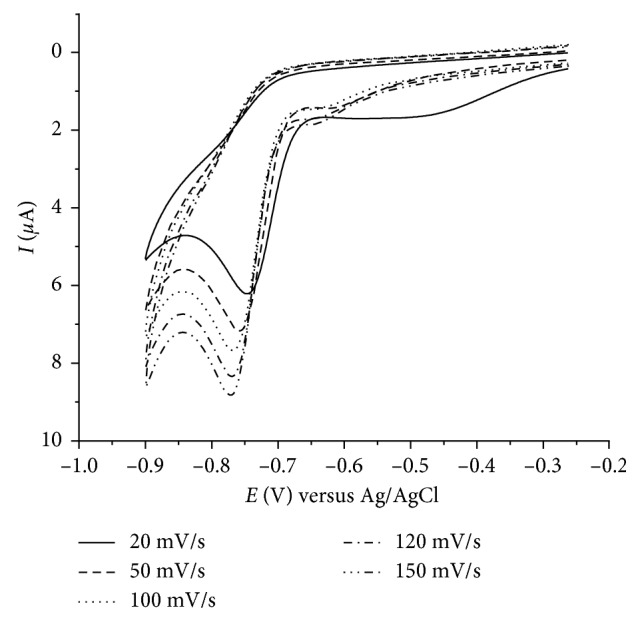
Cyclic voltammograms of 0.5 mol/dm^3^ diazepam on glassy carbon electrode as a function of a scan rate (20–150 mV s^−1^).

Cyclic voltammograms of a 0.5 mol/dm^3^ diazepam in HCl/KCl buffer (pH 1) were recorded in the potential range of −0.2 to −0.9 V. Voltammograms were recorded for different scan rates from 20 to 150 mV s^−1^. In the negative scan, one reduction peak can be observed at around −0.76 V (Figure 1). The value of the peak potential became more cathodic with increasing scan rate and varied linearly with square root of scan rate, indicating irreversibility of the reduction and diffusion-controlled process on the electrode surface.

**Figure 2:**
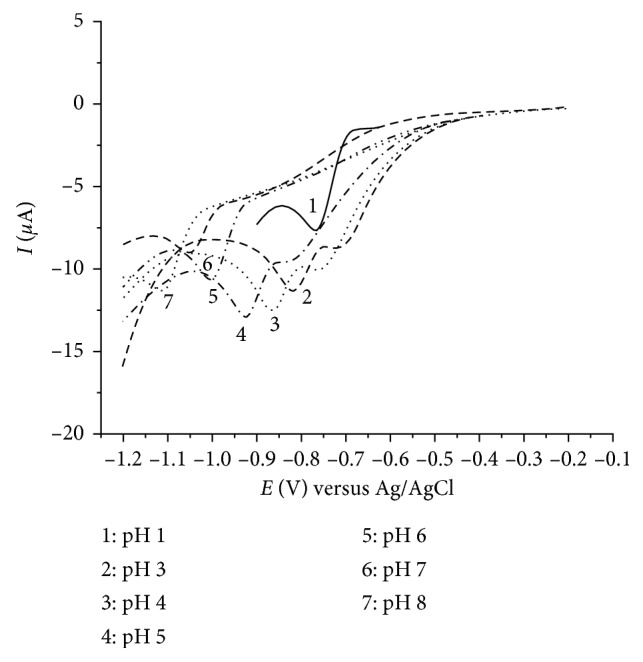
Cyclic voltammograms of 100 *µ*mol/dm^3^ diazepam as function of pH in HCl/KCl buffer (pH 1) and Britton–Robinson buffer (pH 3–8) at 100 mV s^−1^ scan rate.

The effect of pH on reduction of diazepam was investigated when 100 *µ*mol/dm^3^ diazepam was recorded in HCl/KCl buffer (pH 1) and Britton–Robinson buffer of different pH (3–8) values in the potential range −0.2 to −1.2 V. With increase of pH, the cathodic peak shifts to more negative values indicating the dependence of the reduction potential on the pH value of the medium (Figure 2). As the media becomes less acidic, the FIA signal becomes less stable and reproducible. Hence, the further experiments were performed in pH 1 solutions.

The cathodic peak is a result of a two-electron change, and the reduced product can be characterised as 4,5-dihydro-diazepam (Reaction (1)), as reported elsewhere [22, 26]:

(1) $$\begin{array}{c} \text{R}–\left(\text{H}\right)\text{C}=\text{N}–{\text{R}}^{\prime }+2e^{-}{+2\text{H}}^{+}\to \text{R}–\text{C}{\left(\text{H}\right)}_{2}–\text{N}\left(\text{H}\right)–{\text{R}}^{\prime } \end{array}$$

### 3.2. Optimization of Flow Injection Parameters

The following parameters were investigated for the sensitivity of the thin-layer flow cell: the volume of the sample loop and the gasket thickness. The optimal conditions in FIA system were investigated with 100 *µ*mol/dm^3^ diazepam solution. Sample loops of 0.050, 0.075, and 0.120 cm^3^ were tested. The detector response was the most sensitive with 0.050 cm^3^ of the injected sample. The gasket determines the working volume of the flow cell. All four commercially available gaskets from BASi were tested (13, 51, 127, and 381 *µ*m thickness). For this type of flow cell, the best sensitivity was obtained for 51 *µ*m thick gasket, which was already confirmed in our previous work [25].

### 3.3. Optimization of Differential Pulse Amperometry

**Figure 3:**
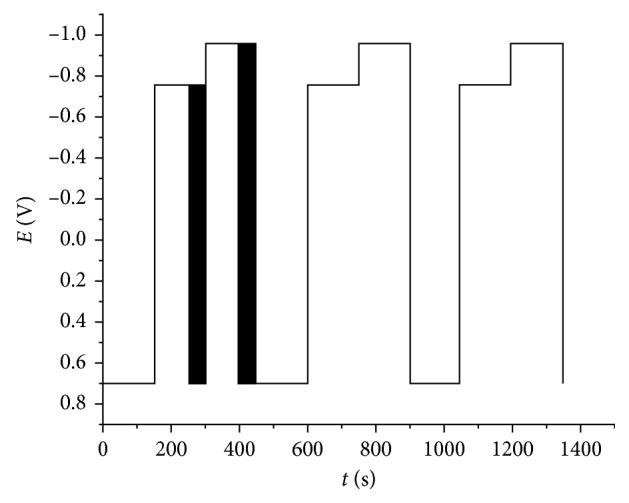
Schematic presentation of DPA waveform applied for diazepam determination. The squares are used for current measurement.

The potentials used for the two DPA pulses were chosen according to the results obtained by cyclic voltammetry. Hence, the DPA pulses should be a little lower and a little higher than the peak potential of diazepam reduction. We investigated the following values of potential pulses, *E*_clean_ 0 to +1 V, *E*_1_ −0.6 to −0.75 V, and *E*_2_ −0.8 to −0.95 V; each potential was held for 50 ms, and the current was collected at last 25 ms for *E*_2_ and *E*_1_. All experiments were performed with 0.1 mmol/dm^3^ diazepam solution in HCl/KCl buffer pH 1. The 0.7 V was applied for cleaning potential due to the signal shape and the system reproducibility. The sensitivity of the detector for different values of *E*_1_ and *E*_2_ is presented in Table 1, and it shows that the best response was obtained for *E*_1_ = −0.75 V and *E*_2_ = −0.95 V.  Figure 3 presents the DPA waveform applied for diazepam.

Under these conditions, there was no need for purging the solutions prior the experiments or working in the inert atmosphere.

### 3.4. Analytical Parameters

**Figure 4:**
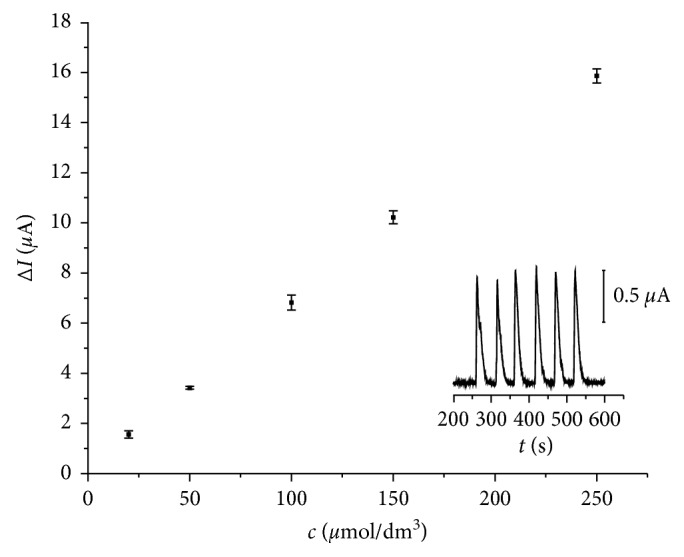
Linear response for FIA-DPA system for 20–250 *µ*mol/dm^3^ diazepam. Inset: six consecutive injections of 100 *µ*mol/dm^3^ of diazepam.

The calibration curve obtained from the DPA peak current for diazepam reduction at different diazepam concentration shows linear behaviour between 20 and 250 *µ*mol/dm^3^ with the regression equation Δ*I* (*µ*A) = 0.063*c* (*µ*M) + 0.430 (Figure 4). The presented calibration curve is the average obtained from the three curves recorded for the same set of standards; error bars in each point of the curve are standard deviations. The detection limit was calculated to be 13.4 *µ*mol/dm^3^, but measured as 3 *s*/*m*, where *s* is the standard deviation and *m* is the slope, and the limit of quantification was calculated to be 44.8 *µ*mol/dm^3^. Obtained limit of the detection is not comparable to LODs obtained by hyphenated techniques (chromatography/mass spectrometry) which often require complicated sample preparation [13, 27–30]. Electrochemical detection is seldom described for determination of diazepam in beverage samples. Honeychurch et al. [20] applied adsorptive stripping voltammetry with medium exchange limit, and the limit of detection was 1.8 *µ*g cm^−3^.

The absolute limit of detection for the sample loop volume of 0.050 cm^3^ was 0.19 *µ*g of diazepam. Reproducibility of the system was checked by six consecutive injections of 100 *µ*mol/dm^3^ DZP on the same day for investigation of intraday precision (insert picture on Figure 4), and the relative standard deviation for intraday precision was 1.53%. Interday precision was obtained by recording peak current of 100 *µ*mol/dm^3^ DZP during three consecutive days; the results showed decrease of precision, presented by the relative standard deviation of 10.8% for interday measurements. Since simple flow injection system was used and the signal frequency was dependent on tubing length, only the throughoutput was up to 90 analyses per hour (Table 2).

### 3.5. Interference Study

Ascorbic acid, lactose, glucose, and citric acid are the common compounds present in the beverage samples. The effect of interferants on diazepam determination was investigated by injecting solutions containing 100 *µ*mol dm^−3^ of each interferant and mixtures with diazepam of the same concentration. Results showed that interferants decrease diazepam signal by 4–10% (Table 3).

### 3.6. Determination of Diazepam in Beverage Samples

The purpose of this method was to investigate concentrations relevant for forensic studies. On Serbian market, diazepam tablets containing 2, 5, or 10 mg of active substance are available; when a tablet is dissolved in 200 cm^3^ of a beverage, obtained concentrations are from 35–175 *µ*mol/dm^3^ of diazepam, which fits in the linear range of the detector.

Beverage samples (Guarana and two beer samples) were purchased at a local store. 12.5 cm^3^ of a sample was diluted with buffer and appropriate volume of diazepam standard solution, ultrasonicated for 30 minutes, and made up with distilled water to 25.00 cm^3^. The samples were spiked with 50 *µ*mol/dm^3^ of diazepam. Solutions were injected in triplicate, and the results of recoveries for spiked samples of different concentrations are presented in Table 4.

## 4. Conclusions

Flow injection method with differential pulse amperometry was developed and applied for quantification of diazepam in investigated set of beverage samples. Differential pulse amperometry enables the determinations in the presence of oxygen without surface modification. Under the optimal conditions, the linear range of the system was from 20 to 250 *µ*mol/dm^3^ of diazepam, with the detection limit of 3.83 *µ*g/cm^3^, the intraday precision of 1.53%, and a throughoutput of 90 analyses per hour. The method was applied on diazepam determination in beverage samples; 0.050 cm^3^ of sample was enough for fast, precise, and sensitive quantification. With the flow rate of 1 cm^3^/min, the method is economical, producing small volumes of waste. The simple sample preparation enables injection of samples without extraction or filtration.

## Figures and Tables

**Table 1:** Sensitivities at different *E*_1_ and *E*_2_ values for FIA-DPA.

*E*_1_ (V)	*E*_2_ (V)	Slope (nA dm^3^ mol^−1^)
−0.6	−0.8	35
−0.65	−0.8	42
−0.7	−0.8	31.3
−0.75	−0.8	14.2
−0.75	−0.9	38.8
−0.75	−0.95	92.7

**Table 2:** Analytical characteristics of the proposed methods.

Characteristics	FIA-DPA
LR, *µ*mol dm^−3^	20–250
Sensitivity, nA dm^3^ *µ*mol^−1^	63
Correlation coefficient, *r*	0.997
LOD, *µ*mol dm^−3^	13.4
Intraday RSD (*n*=6)	1.53%
Interday RSD (*n*=3)	10.8%
Throughoutput, 1 h^−1^	90

**Table 3:** Results of interference study.

Interferent	Relative signal decrease (%)
Citric acid	4
Lactose	5
Ascorbic acid	8
Glucose	10

**Table 4:** Analysis results of the recovery for spiked samples (*n*=3).

Sample	Spiked concentration (*µ*mol/dm^3^)	Results (mean ± SD) (*µ*mol/dm^3^)	Recovery ratio (%)
Guarana	50	52.48 ± 0.80	104.96
	100	101.25 ± 1.55	101.25
	150	152.05 ± 2.33	101.37
Beer I	50	47.51 ± 0.73	95.02
	100	94.31 ± 1.44	94.31
	150	139.48 ± 2.13	92.99
Beer II	50	49.12 ± 0.75	98.24
	100	96.24 ± 1.47	96.24
	150	141.66 ± 2.17	94.44
